# Opportunistic myiasis is a rare complication in patients with feeding gastrostomy tube

**DOI:** 10.1055/a-2513-2804

**Published:** 2025-02-05

**Authors:** Ayman Alsebaey, Hassan Abdullah Alsolami, Salem Mohamed Bafarag, Amr Ibrahim Issa, Fahd Mohammed Almalki

**Affiliations:** 1 68873 Department of Hepatology and Gastroenterology, Menoufia University National Liver Institute, Shebeen El-Koom, Egypt; 2 Department of Gastroenterology, King Faisal Hospital, Ministry of Health, Mecca, Saudi Arabia; 3 473047 Department of Medicine, College of Medicine, Umm Al-Qura University, Mecca, Saudi Arabia

A 55-year-old man who suffered from cancer of the larynx was referred 6 months ago for endoscopic percutaneous gastrostomy tube (PEG) for feeding. The PEG was placed using an ultrathin scope to pass the throat mass. The patient received multiple chemotherapy and radiotherapy sessions. The patient’s family came to the emergency room when they noticed worms in his undershirt. The abdomen was lax on examination, and on removal of the old dirty dressing, a lot of maggots were seen moving around the gastrostomy tube ( Fig. 1 **,** Video 1 ). Mild cellulitis was noted in the surrounding area. The dermatologist and surgeon were consulted, and irrigation, larvae extraction, and a petroleum jelly dressing were applied successfully. Topical antibiotics were prescribed. A new replacement gastrostomy was performed successfully. Family education was provided and good hygiene encouraged.

Maggots around the gastrostomy tube.Video 1

**Fig. 1:**
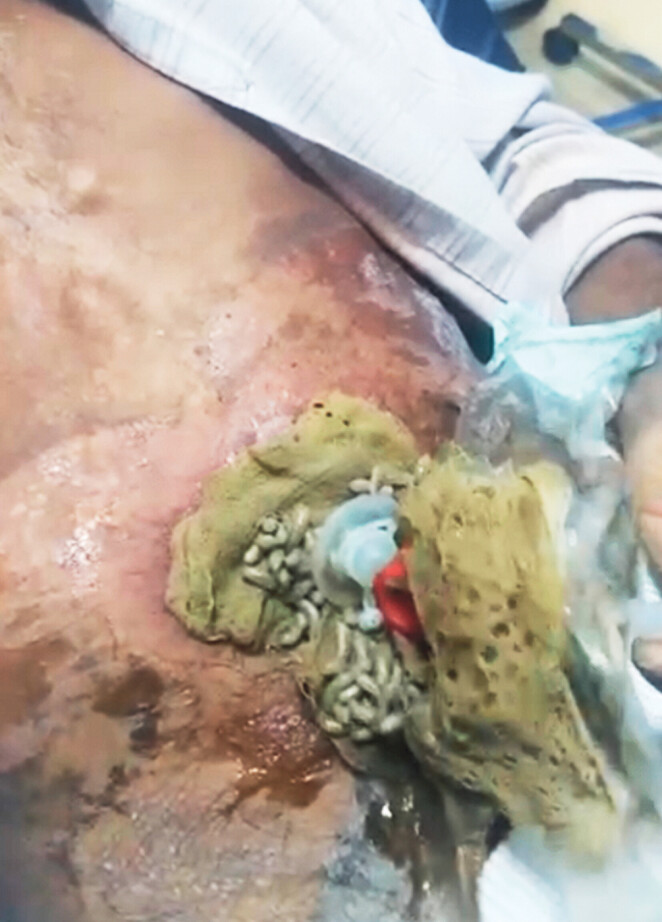
Maggots around the gastrostomy tube.

Myiasis is an unusual maggot (larval) infestation of living organisms. Clinically there are two types, namely “obligate,” which is travel-associated, and opportunistic “facultative or traumatic,” which is commonly seen in necrotic wounds 1 . Obligate myiasis is caused by two flies: *Dermatobia hominis* (bоtfly) and *Cordylobia anthropophaga* (tumbu fly). The larvae commonly penetrate the skin and the underlying tissue. Usually, the insect bite progresses to a nodule that may ooze serosanguineous fluid, causing skin irritation and itching. Myiasis may be complicated by secondary bacterial infection 2 .

Wound myiasis is caused by the *Calliphoridae* (blowfly or screwworm fly), *Sarcophagidae* (flesh fly), and *Phoridae* (humpback fly) families. The condition is common with poor personal hygiene. The common treatment is irrigation to remove the dislodged larva, larvae removal, closure of the skin openings by an occlusive dressing, petroleum jelly and submersion using diluted povidone-iodine solution 3 .

In the literature, two cases were published reporting myiasis around the percutaneous gastrostomy tube 4 5 . The first case was a 71-year-old suffering from diabetes mellitus, hypertension, and an old stroke who needed a feeding gastrostomy tube. The other case was a 55-year-old with metastatic squamous cell carcinoma who needed a feeding gastrostomy tube. In conclusion, myiasis is an uncommon complication of a gastrostomy tube in a patient with poor hygiene.

Endoscopy_UCTN_Code_CPL_1AH_2AI

## Notes

Endoscopy E-Videos https://eref.thieme.de/e-videos
*E-Videos* is an open access online section of the journal *Endoscopy* , reporting on interesting cases and new techniques in gastroenterological endoscopy. All papers include a high-quality video and are published with a Creative Commons CC-BY license. Endoscopy E-Videos qualify for HINARI discounts and waivers and eligibility is automatically checked during the submission process. We grant 100% waivers to articles whose corresponding authors are based in Group A countries and 50% waivers to those who are based in Group B countries as classified by Research4Life (see: https://www.research4life.org/access/eligibility/ ). This section has its own submission website at https://mc.manuscriptcentral.com/e-videos .
